# The evolutionary advantage of guilt: co-evolution of social and non-social guilt in structured populations

**DOI:** 10.1098/rsif.2025.0164

**Published:** 2025-07-30

**Authors:** Theodor Cimpeanu, Luis Moniz Pereira, The Anh Han

**Affiliations:** ^1^ Department of Biological and Environmental Sciences, University of Stirling, Stirling, UK; ^2^ Department of Computer Science, Universidade Nova de Lisboa, Lisbon, Portugal; ^3^ SCEDT, Teesside University, Middlesbrough, UK

**Keywords:** evolution of cooperation, social dilemma, guilt, emotion modelling, evolutionary game theory, structured populations, population dynamics, complex networks, agent-based modelling, ethical AI

## Abstract

Building ethical machines may involve bestowing upon them the emotional capacity to self-evaluate and repent for their actions. While apologies represent potential strategic interactions, the explicit evolution of guilt as a behavioural trait remains poorly understood. Our study delves into the co-evolution of two forms of emotional guilt: social guilt entails a cost, requiring agents to exert efforts to understand others’ internal states and behaviours; and non-social guilt, which only involves awareness of one’s own state, incurs no social cost. Resorting to methods from evolutionary game theory, we study analytically, and through extensive numerical and agent-based simulations, whether and how guilt can evolve and deploy, depending on the underlying structure of the systems of agents. Our findings reveal that in lattice and scale-free networks, strategies favouring emotional guilt dominate a broader range of guilt and social costs compared to non-structured well-mixed populations, leading to higher levels of cooperation. In structured populations, both social and non-social guilt can thrive through clustering with emotionally inclined strategies, thereby providing protection against exploiters, particularly for less costly non-social strategies. These insights shed light on the complex interplay of guilt and cooperation, enhancing our understanding of ethical artificial intelligence.

## 1. Introduction

We are guilty for no reason, or just because we exist anyway, and are imperfect.

Peter J. Conradi [1].

Machine ethics, focused on the potential for artificial intelligence (AI) to engage in moral conduct, represents an interdisciplinary open project for scientists and engineers [2–5]. An essential challenge within this field is the development of effective ways to represent emotions, like guilt, which are thought to shape human moral behaviour, in computational models [6–11]. Upon introspection, interpersonal guilt is present as a feeling of being worthy of blame for a moral offence committed against others. Carrying the burden of guilt, a person may subsequently work towards restoring an internal state untainted by blame, ensuring the absence of this distressing emotion [12]. The popular trend in research is to consider guilt more than shame as leading to reparative actions. This has been looked at in [13], stating that guilt entails reparative action when there is a conscious admission and accountability of the wrongdoing by the transgressor.

Sociocentric and egocentric cultures supposedly have different emotional expressions and experiences of shame and guilt. Sociocentric cultures, which are more social-looking, tend to generate more of a sense of character-intrinsic general shame, while more individualistic egocentric cultures lead to a sense of specific action-intrinsic guilt in the transgressor [14]. While shame and guilt are commonly thought to be synonymous, shame is defined as a self-centric emotion prompting the desire to hide and escape, whereas guilt is characterized by the motivation to engage in reparative actions [15,16]. One feels guilt for having told a lie, but one feels shame for being a liar [17, p. 102]). Guilt concerns transgressions; shame involves shortcomings. Guilt urges reparative action; shame encourages social withdrawal. Guilt requires that a person believes they deserve punishment—even self-inflicted—and that others who are guilty also deserve it.

When norms are well-established, societal members accept them as mandatory, internalize and comply with them and experience guilt or shame when violating them. When internal sanctions do not support compliance over extended periods of time, external sanctions may be necessary [18].

Tomasello [19] emphasized that prior joint objective commitment, or even a subjective commitment, makes guilt feel deserved. Guilt is a process of socially normative self-regulation. Besides self-punishment, there is a sense of ‘I ought not to have done that’ implied in feeling guilty, be it even because of a prior commitment. There is a normative, not just a strategic force in guilt, conducive to repairing damage, with a concern to maintain one’s cooperative identity. The conviction that one should prioritize doing the right thing has the potential to supersede self-centred motivations, extending beyond mere strategic reputation management. Guilt does not necessarily stem from a breach of any conventions; it is not about feeling remorse solely for non-conformity itself. Rather, guilt is selectively aimed at one’s previous judgement of moral rightness: ‘I thought at the time it was the right thing to do but, now aware of the consequences, I no longer do so’. The overt response to guilt is thus to make reparations for harm done. The fact that guilt is a judgement about one’s previous judgement comes out clearly in the fact that humans quite often feel the need to display their guilt overtly, in everything from body postures to verbal apologies. This display may pre-empt punishment from others and may additionally be seen as strategic because it shows solidarity with those who can judge them harshly, and indeed, that it is accepted the negative judgement is deserved and legitimate. Reflective endorsement and guilt, therefore, represent a new kind of social self-regulation, an internalized and reflective self-regulation comprising multiple levels of moral judgement. Violators of moral norms punish themselves through feelings of guilt. They take on the perspective and attitude of the group when judging what they themselves have done.

Guilt, as an emotion, incorporates a cognitive component, involving the acknowledgement that the subject has, in some way, violated a norm (survivor guilt being an exception) [20, p. 196]. There is even the potential for feeling guilty about the absence of guilt—a meta-emotion [21, p. 404]. The anticipation of guilt can drive normative conformity, even in the absence of an expected retaliatory response. When we anticipate the wrath of others or our own guilt, this can defeat the temptation to engage in subsequent harmful behaviour. Guilt promotes cooperative behaviour by adding an emotional cost to defection [22, p. 141]. Reciprocity violations provoke one’s anger and an other’s guilt [21, p. 380]. Self-directed anger plays a different role than guilt. People get angry at themselves when they behave stupidly, but guilt arises when people violate norms (or commitments) and is especially likely when one causes harm to others. In addition, we feel more guilty about harming members of the in-group than those of the out-group. This finding leads some authors to conclude that guilt is an emotion that arises especially when there is a threat of separation or exclusion [22, p. 133]. Moreover, it highlights the importance of population networking with respect to guilt.

Guilt-proneness has been highlighted as independent of anger; in other words, individuals with a predisposition to guilt are no more or less prone to anger than the general population [12, pp. 490−496]. Yet, when confronted with anger, those with a propensity for guilt are more likely to channel it constructively, opting for non-hostile discussions, direct corrective actions and a general aversion to aggression. Feeling guilty leads to positive intrapersonal and interpersonal processes. Expressions of guilt can strengthen relationships in a number of ways, especially in contexts requiring cooperation and interpersonal trust, based on assumptions of equity and fairness.

Guilt triggers self-debugging, as a result of an *a posteriori* error detection in norm compliance. There is an expectation of correctness and a dissonance provoked by error, debugging being enabled by counterfactual reasoning [23,24].

Righteousness is arguably the opposite of guilt [25, p. 17]. Guilt, arising from rule violation, contrasts with righteousness, a rewarding state achieved through rule adherence. When individuals ‘do the right thing’, they experience a distinct positive emotion. Righteousness acts as a proxy for the rewards of conformity and serves to encourage it. Righteous individuals are willing to pay a price for resisting the temptation to swindle others.

In social dilemma games such as the prisoners’ dilemma (PD) [26], where defection or cheating becomes the dominant strategy, defectors do better than cooperators regardless of whether their partners defect or cooperate [27]. In such a situation, it is rational for both parties to defect, even though mutual defection is mostly overall worse than reciprocal cooperation. Trivers [28] speculated that mutual evolution has promoted the emergence of guilt because it makes defection less attractive, with motivation from guilt becoming the dominant strategy due to attending social benefits. Individuals may gain materially by defecting, but guilt causes emotional suffering, and it is this suffering avoidance that encourages cooperation regardless of material gain. Nesse [29] sustains that the temptation to defect arouses anxiety and defection arouses guilt, both aversive emotions that inhibit hasty selfishness. Guilt will motivate apologies and/or self-punishment otherwise, and reparations are needed to re-establish trust. Using an iterated PD (IPD) game, Ketelaar & Tung Au [30] found that inducing guilt increased cooperativeness among previously uncooperative players.

From an evolutionary viewpoint, guilt is envisaged as an in-built mechanism that tends to prevent wrongdoing. Internal suffering and the need to alleviate it press an agent to their admission after wrongs are enacted, involving costly apology or penance, a change to correct behaviour, and an expectation of forgiveness to dispel the guilt-induced suffering. The hypothesis then is that within a population, the emergence of guilt and its effects are evolutionary advantageous compared to a guilt-free population. Moreover, the magnitude of the advantage presumably depends on the population’s actual network structure, since it governs who is in touch with whom and affected by whom [31,32] and determines the extent to which the social costs of guilt are globally worthwhile.

Inspired by the discussed psychological and evolutionary studies of guilt and cooperation in networks [31–38], here we provide a theoretical account of the evolution of costly guilt-prone behaviours in the context of distributed multi-agent systems (MAS), with the overarching aim of achieving new insights for the design and engineering of cooperative, self-organized systems. Resorting to methods from evolutionary game theory (EGT) and agent-based simulations [27,39], we study the evolution of social versus non-social aware guilt in differently structured populations.

We shall examine whether (non-)social guilt can evolve in such structured populations, e.g. through the clustering of similarly emotionally prone individuals. Social guilt, and social emotions in general, depend upon awareness of the thoughts, feelings or actions of others in the environment [40,41]. Thus, choosing to be social can be (much) more costly compared to being non-social, requiring efforts to understand or be more aware, through observation of others’ thoughts and feelings and the context behind their actions, while non-sociality only requires awareness of one’s own internal physical state. Non-social guilt is an internal mechanism*—I* did something bad to another; therefore, *I* feel suffering as a consequence. This requires no awareness of the other’s emotional state. Social guilt is more akin to a norm. I only feel guilt if the other person is also repentant. In other words, I only feel guilt if they also feel when they have broken a norm so that there is something for me to feel guilty about. Hence, one might inquire whether and when such a more cost-efficient non-social strategy can evolve (though more easily exploitable as we will see), depending on the specific underlying network structure.

Herein, we fundamentally extend and generalize the work we set forth in [10], which constructed theoretical models representing guilt to study its role in promoting pro-social behaviour, in the context of EGT using the IPD. Guilt was modelled in terms of two joint features. Firstly, guilt involving a record of transgressions formalized as a counter-tracking the number of offences. Secondly, guilt involving a threshold over which the guilty agent must alleviate its strained internal state, by means of deliberate change of behaviour plus self-punishment, as required by the negative feelings of guilt, changes such that would affect the game’s payoff for the guilty party.

## 2. Models and methods

Firstly, we recall the IPD game and the definition of guilt-prone strategies, as described in [10]. Next, we describe our model where social and non-social guilt strategies are in co-presence. Then, the methods for analysing the model, namely stochastic evolutionary dynamics in well-mixed populations and agent-based simulations in networks, are in turn detailed.

### 2.1. Iterated prisoners’ dilemma

In each round of the IPD, two players engage in a PD game interaction where its outcomes are defined by the following payoff matrix (for the row player):


$$\begin{array}{c} C\\ D \end{array}\begin{array}{l} \;C\;D\\ \left(\begin{array}{cc} R & S\\ T & P \end{array}\right). \end{array}$$

A player who chooses to cooperate (*C*) with another who defects (*D*) receives the sucker’s payoff S , whereas the defecting player gains the temptation to defect, T . Mutual cooperation (resp., defection) yields the reward R (resp., punishment *P*) for both players. Depending on the value ordering of these four payoffs, different social dilemmas arise [26,27]. In this work, we are namely concerned with the PD, where T>R>P>S . In a single round, it is always best to defect, because less risky, but cooperation may be rewarding if the game is iterated. In the IPD, it is also required that mutual cooperation be preferred over an equal probability of unilateral cooperation and defection (i.e. 2R>T+S ), since otherwise, alternating between cooperation and defection would lead to a higher payoff than mutual cooperation. The PD is repeated for a number of rounds, Ω .

For a convenient interpretation of results, we also consider the simplified version of the PD, the Donation game [27], where the payoff entries are specifically described via the cost c ( c>0 ) and benefit b ( b>c ) of cooperation, as follows: T=b , R=b−c , P=0 , S=−c .

### 2.2. Guilt modelling in iterated prisoners’ dilemma

We base our model and analysis on the approach set forth in [10], which formalizes guilt as an aspect of an agent’s genotypical strategies and is quantified in terms of a threshold, *G*. In this model, G∈[0,+∞] and guilt at any given time is characterized by a transient level of guilt, g ( g≥0 ). As the experiment begins, g for every agent is set to 0 . It increases by 1 after each action that the agent considers wrong. After several accumulated wrongdoings result in g reaching that agent’s threshold of guilt, g≥G , the agent can choose to (or not to) act to reduce its guilt level g below that threshold. The model retains the mechanism of guilt alleviation described above, whereby guilt can be alleviated by apologizing to offended partners, or by suffering guilt through self-punishment whenever apology to offended partners is not an option. In the sequel, we will suppose the latter case. Either way, the guilty party suffers a cost. Indeed, the alleviation of guilt is costly, this cost being quantified in terms of γ ( γ≥0 ), whenever g is decreased by 1 . In accordance with this definition, agents can be characterized with respect to different guilt thresholds. Some may be incapable of suffering guilty feelings, meaning their G=+∞ . Others may be extremely prone to guilt, suffering guilty feelings with any first mistake, so for them G=0 . These are the only two cases to be considered below.

### 2.3. Social versus non-social guilt in co-presence

In this setting, a strategy is described by three factors or components.

#### 2.3.1. Guilt threshold

Since we shall focus in the current work on understanding the evolution of social guilt behaviours, or their absence, as well as the impact on them of network structures, we consider the following two basic types of guilt thresholds:

—
G=+∞ : in this type of agent, the guilt level g will never reach the threshold no matter how many times they defect; hence, they never need to reduce g , and consequently never pay the guilt cost γ . In other words, this type of agent experiences no guilt feeling. They are dubbed (guilt-)unemotional agents.
—
G=0 : whenever this type of agent defects, it becomes immediately true that g>G ; hence, the agent needs to act immediately to reduce g , by paying γ . In other words, this type of agent always feels guilty subsequent to just a single wrongdoing, i.e. defection. They are dubbed (guilt-)emotional agents.

#### 2.3.2. Decision making in the iterated prisoners’ dilemma

An agent can choose to play either *C* or *D* in a PD, and, given the agent’s guilt threshold G , if its ongoing guilt level g reaches G , they can choose whether to change their behaviour from *D* to *C* (to avoid further emotional pain and cost).

#### 2.3.3. Sociability versus non-sociability about when to feel guilty

**Figure 1.**
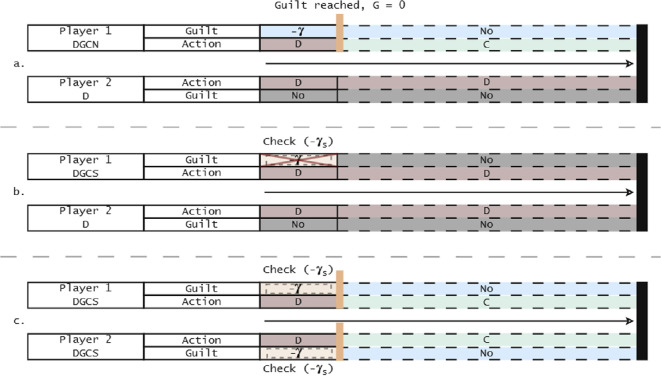
Diagrams representing repeated interactions between emotionally prone players. In (a), an emotionally adaptive non-social defector interacts with another defector; they feel guilty after one interaction (here G=0 ) and change their behaviour to prevent further internal pain. In (b), an emotionally adaptive social defector checks whether their partner felt guilt for their actions, and, if so, does not feel guilt nor change behaviour in future interactions. In (c), two of the adaptive social players interact; after checking, they feel guilty for their first transgressions, and so cooperate in future interactions.

The emotional agents can choose to be non-social or social, regarding the way they express their emotions. To be social, agents need an extra effort such as signalling their guilt or observing the co-player’s guilt, as this observation will affect how they themselves will react (see figure 1 for a visual representation of interactions with social and non-social strategists). Hence, we assume there is always an additional cost, $\gamma_{s}$ , to being social.

Overall, since we do not yet consider noise in IPD (i.e. non-deliberate mistakes) in this work, there are in total six possible strategies,^1^ denoted as follows:

(1) Unemotional cooperator (*C*): always cooperates (*C*), unemotional (i.e. G=+∞ ). Does not feel guilt, does not change behaviour.
(2) Unemotional defector (*D*): always defects (*D*), unemotional (i.e. G=+∞ ). Does not feel guilt, does not change behaviour.
(3) Emotional non-adaptive defector that is non-social (DGDN): always defects (*D*), feels guilty (*G*) after one wrongdoing (i.e. G=0 ), does not change its behaviour (thereby the second *D*), regardless of what the co-player feels (hence its non-sociability *N*).
(4) Emotional adaptive defector that is non-social (DGCN): defects initially (*D*), feels guilty (*G*) after one wrongdoing (i.e. G=0 ), changes its behaviour from *D* to *C* (hence the *C*), regardless of what the co-player feels (hence its non-sociability *N*).
(5) Emotional non-adaptive defector that is social (DGDS): always defects (*D*), feels guilty (*G*) after one wrongdoing (i.e. G=0 ) but only if their co-player also feels guilty after a wrongdoing (hence its sociability *S*), but does not change its behaviour (hence the second *D*).
(6) Emotional adaptive defector that is social (DGCS): defects initially, feels guilty after one wrongdoing (i.e. G=0 ) but only if their co-player also feels guilty after a wrongdoing (hence its sociability *S*), and changes its behaviour from *D* to *C* (hence the *C*).

From the above costs, we can derive the payoff matrix for these six strategies (for the row player), as follows:


(2.1) $$\begin{array}{c} 1\\ 2\\ 3\\ 4\\ 5\\ 6 \end{array}\begin{array}{l} \;C\;D\;\text{DGDN}\;\text{DGCN}\;\text{DGDS}\;\text{DGCS}\\ \left(\begin{array}{cccccc} R & S & S & \frac{S+R\Theta }{\Omega } & S & \frac{S+R\Theta }{\Omega }\\ T & P & P & \frac{P+T\Theta }{\Omega } & P & P\\ T-\gamma & P-\gamma & P-\gamma & \frac{P+T\Theta }{\Omega }-\gamma & P-\gamma & \frac{P+T\Theta }{\Omega }\\ \frac{T-\gamma -\gamma_{s}+R\Theta }{\Omega } & \frac{P-\gamma -\gamma_{s}+S\Theta }{\Omega } & \frac{P-\gamma -\gamma_{s}+S\Theta }{\Omega } & \frac{P-\gamma -\gamma_{s}+R\Theta }{\Omega } & \frac{P-\gamma -\gamma_{s}+S\Theta }{\Omega } & \frac{P-\gamma -\gamma_{s}+R\Theta }{\Omega }\\ T-\gamma -\gamma_{s} & P-\gamma_{s} & P-\gamma -\gamma_{s} & \frac{P+T\Theta }{\Omega }-\gamma -\gamma_{s} & P-\gamma -\gamma_{s} & \frac{P+T\Theta }{\Omega }-\gamma -\gamma_{s}\\ \frac{T-\gamma -\gamma_{s}+R\Theta }{\Omega } & P-\gamma_{s} & \frac{P-\gamma -\gamma_{s}+R\Theta }{\Omega } & \frac{P-\gamma -\gamma_{s}+R\Theta }{\Omega } & \frac{P-\gamma -\gamma_{s}+R\Theta }{\Omega } & \frac{P-\gamma -\gamma_{s}+R\Theta }{\Omega } \end{array}\right), \end{array}$$

where we employ Θ=Ω−1 just for the purpose of neater representation.

In order to understand when guilt can emerge and promote cooperation, our EGT modelling study below analyses whether and when emotional strategies, i.e. those with G=0 , can actually overcome the disadvantage of the incurred costs or fitness reduction associated with the guilt feeling and its alleviation and, as a consequence, be able to disseminate throughout the population.

Previous work shows that some emotional guilt-based responses only make sense when the co-player is not attempting to harm you too, or else attempting to harm you but feeling guilty as well [10]. That is, guilt needs to be social to prevail in social dynamics. The main reason is that players who feel guilty after a wrongdoing, regardless of others’ behaviours (i.e. whether these others signal guilt or else are observed to feel guilty), would be exploited by non-emotional defectors (i.e. the *D* strategy). We argue that, since being social is costly, since agents need to observe and understand others’ actions and feelings, non-social guilt might conceivably be more cost-efficient and prevail in network environments where they might be protected from such *D* strategy exploiters by non-connection. Because previous guilt modelling work only looked at well-mixed networked populations, wherein all individuals in the population interact with one another, it was not possible to consider such eventual network connectivity protection. To bridge this gap, in this work, we shall address several cases of structured populations, wherein players interact only with their direct neighbours.

### 2.4. Evolutionary dynamics in well-mixed populations

In our analysis, individuals’ payoffs signify their *fitness* or social *success*, and evolutionary dynamics is shaped by social learning [27,42]. In this process, the agents who achieve higher success are more likely to be imitated by their others. In the current work, social learning is characterized by the so-called pairwise comparison rule [43], a common approach in EGT. This rule assumes that an agent A with a fitness value $f_{A}$ adopts the strategy of another agent B with a fitness value $f_{B}$ with a probability p determined by the Fermi function


(2.2) $$p_{A,B}={\left(1+{\text{e}}^{-\beta (f_{B}-f_{A})}\right)}^{-1}.$$

The parameter β denotes the ‘imitation strength’ or ‘intensity of selection’, signifying how strongly agents base their decision to imitate on the fitness difference between themselves and their opponents. When β=0 , the system reaches the limit of neutral drift, where imitation decisions are entirely random. As β increases, imitation becomes increasingly more deterministic. Consistent with previous works and human behavioural experiments [44–46], we adopt β=1.0 in the main text, which also allows us to compare directly with the previous guilt model of [10].

**Figure 2.**
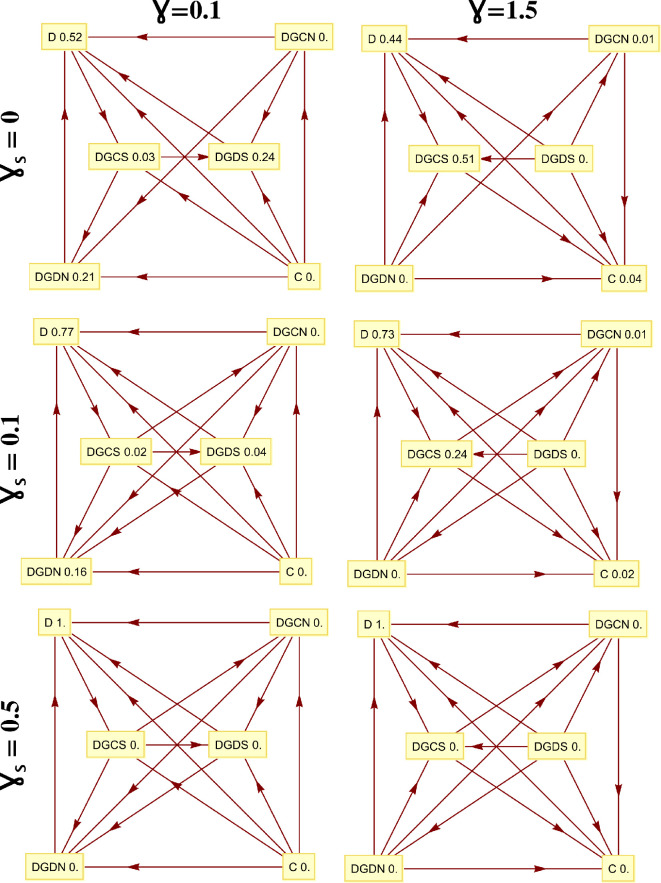
Markov diagrams and stationary distributions (well-mixed populations). Transitions direction among strategies, where the arrows show the direction where the transition probability is stronger than the reverse. The results are in line with risk-dominance analysis (in §3.1). Other parameters: N=100 , Ω=10 , R=1 , S=−1 , T=2 , P=0 .

In the absence of mutations or strategy exploration, the end states of evolution are inevitably monomorphic. Once such a state is attained, it cannot be escaped through imitation. To account for this, we introduce the assumption that, with a certain mutation probability, an agent may switch randomly to a different strategy without the necessity of imitating another agent. In the limit of small mutation rates, it is established that the dynamics will proceed with, at most, two strategies in the population. As a result, the behavioural dynamics can be succinctly delineated through a Markov chain, where each state represents a monomorphic population. In this process, the transition probabilities are given by the so-called fixation probability of a single mutant [47,48]. The resulting Markov chain has a stationary distribution, which characterizes the average time the population spends in each of these monomorphic end states (see some examples in figure 2).

Consider a population of size N . Denote $\pi_{X,Y}$ the payoff a strategist X obtains in a pairwise interaction with strategist Y (as defined by the payoff matrices). Assuming at most two strategies in the population, with k agents using strategy A ( 0≤k≤N ) and (N−k) agents using strategy B. Thus, the (average) payoff of the agent that uses A (similarly for B) is


(2.3) $$\begin{array}{lcr} & \begin{array}{rl} \Pi_{A}(k) & =\frac{(k-1)\pi_{A,A}+(N-k)\pi_{A,B}}{N-1}. \end{array} & \end{array}$$

The probability to change the number k of agents using strategy A by ± 1 in each time step is given as [43]


(2.4) $$T^{\pm }(k)=\frac{N-k}{N}\frac{k}{N}{\left[1+{\text{e}}^{\mp \beta [\Pi_{A}(k)-\Pi_{B}(k)]}\right]}^{-1}.$$

Now, the fixation probability of a single mutant with a strategy A in a population of (N−1) agents using B can be written as [43,48]


(2.5) $$\rho_{B,A}={\left(1+\sum_{i=1}^{N-1}\prod_{j=1}^{i}\frac{T^{-}(j)}{T^{+}(j)}\right)}^{-1}.$$

Considering a set {1,...,q} of distinct strategies, these fixation probabilities establish a transition matrix $M=\{T_{ij}{\}}_{i,j=1}^{q}$ of a Markov chain. Here, $T_{ij,j\neq i}=\rho_{ji}/(q-1)$ and $T_{ii}=1-\sum_{j=1,\;j\neq i}^{q}T_{ij}$ . The normalized eigenvector associated with the eigenvalue 1 of the transposed matrix of M yields the stationary distribution described above [47], depicting the relative time the whole population spends adhering to each of the strategies.

#### 2.4.1. Risk-dominance

A crucial approach to comparing two strategies A and B is to ascertain the direction in which the transition is stronger or more probable—whether an A mutant fixates in a population of agents using B ( $\rho_{B,A}$ ) or a B mutant fixates in the population of agents using A ( $\rho_{A,B}$ ). It can be demonstrated that the former is stronger in the limit of large N , if the following (risk-dominant) condition is satisfied [27,48]:


(2.6) $$\pi_{A,A}+\pi_{A,B}>\pi_{B,A}+\pi_{B,B}.$$

### 2.5. Agent-based simulations and network structures

#### 2.5.1. Network topologies

Connections within a network not only signify proximity in terms of interaction (indicating with whom the agents can interact) but also in an observational sense (highlighting whom the agents can imitate). Thus, the network of interactions aligns with the imitation network [49]. As each network type converges at different rates and naturally presents various degrees of heterogeneity, we employ varying population sizes in our experiments to investigate this, while optimizing run time.

Well-mixed populations, where all interact with all, provide a suitable baseline scenario, as no specific heterogeneous interaction structure is present. Considering the realm of structured populations, we take a step further, probing the role of network properties and structural heterogeneity in cultivating the evolution of guilt-prone behaviours. Initially, we examine square lattice (SL) populations of size N=30×30 , employing periodic boundary conditions, a widely adopted population structure in population dynamics and evolutionary games (for a survey, see [31]), wherein each agent can only interact with its four immediate neighbours. While the SL introduces a network structure, it is noteworthy that all nodes within this set-up can be conceptualized as structurally equivalent.

Taking our investigation a step beyond, we explore complex networks in which the network portrays a heterogeneity that mirrors the power-law distribution of wealth (and opportunity) characteristic of real-world settings. The Barabási and Albert (BA) model [50] is one of the most widely adopted models used in the study of such heterogeneous, complex networks. Key features of the BA model include adherence to a *preferential attachment* rule, a low clustering coefficient and a characteristic *power-law degree distribution*. To elucidate the concept of preferential attachment, we outline below the construction process of a BA network.

Starting from a small set of $m_{0}$ interconnected nodes, each new node selects and establishes a link with m older nodes following a probability proportional to their degree (the number of its edges). This process continues until the network reaches the desired size of N . This will produce a network characterized by a power-law distribution, $p_{k}∼k^{-\chi }$ , where the exponent χ is its degree exponent [51]. Notably, the network has a high degree correlation among nodes, featuring a skewed degree distribution with a prolonged tail. A few hubs in the network attract an increasing number of new nodes, which attach as the network grows (in a typical ‘*rich-get-richer*’ scenario). The power-law distribution observed in BA networks mirrors the heterogeneity found in various real-world networks. The average connectivity of the obtained scale-free (SF) network is z=2m . For all of our experiments, we pre-seed 10 different SF networks of size N=1000 and an average connectivity of z=4 , in alignment with the number of neighbours in a SL.

#### 2.5.2. Computer simulations

Initially, each agent is designated as one of the six strategies (i.e. C, D, DGDN, DGCN, DGDS, DGCS), with equal probability. At each time step, each agent plays the PD with its immediate neighbours. The fitness score for each agent is the sum of the payoffs in these encounters. At the end of each step, an agent A with fitness $f_{A}$ chooses to copy the strategy of a randomly selected neighbour agent B with score $f_{B}$ , with a probability given by the Fermi function [31] in equation (2.2). Similar to the well-mixed setting above, we set β=1 in our simulations.

We simulate this evolutionary process until a stationary state or a cyclic pattern is reached. For the sake of a clear and fair comparison, all simulations are run for $10^{6}$ steps. Moreover, for each simulation, the results are averaged over the final $10^{5}$ generations, in order to account for the fluctuations characteristic of these stable states. Furthermore, to improve accuracy, for each set of parameter values, the final results are obtained by averaging 30 independent realizations (20 for SF networks due to computational overheads and the additional pre-seeding of networks, i.e. 200 replicates for SF networks).

## 3. Results

Given the model and methods described above (see table 1 for a summary of the parameters), we first derive analytical conditions for when guilt-prone strategies can be viable and promote the evolution of enhanced cooperation. Next, we obtain simulated numerical results for the well-mixed population setting, validating the analytical conditions. We then show results from our extensive agent-based simulations in structured population settings.

**Table 1.** Model parameters.

parameter	symbol
population size	N
cost of cooperation	c
benefit of cooperation	b
intensity of selection	β
guilt cost	γ
social cost of guilt	$\gamma_{s}$
number of rounds in IPD	Ω
guilt threshold	G

### 3.1. Risk dominance of guilt-prone strategies

To start with, we obtain analytical conditions for when guilt-prone strategies can be evolutionarily viable against other strategies. For that, we apply the risk-dominance criteria in (2.6) to the payoff matrix given in (2.1).

First, DGCS is risk-dominant against DGDS if


(3.1) $$\begin{array}{lcr} & \gamma +\gamma_{s}>\frac{T-R+P-S}{2}=c. & \end{array}$$

The condition for DGCS to be risk-dominant against C is the reverse of that of against DGDS above. DGCS is risk-dominant against DGDN if


(3.2) $$\begin{array}{lcr} & (\Omega -1)\gamma -\gamma_{s}>(\Omega -1)\frac{T-R+P-S}{2}=(\Omega -1)c. & \end{array}$$

It can be seen that this condition subsumes the one for risk-dominance against DGDS above. Also, for this inequality to hold the necessary condition is γ>c .

Now, DGCS is risk-dominant against *D* if


(3.3) $$\begin{array}{lcr} & \gamma +(\Omega +1)\gamma_{s}<(\Omega -1)(R-P)=(\Omega -1)(b-c). & \end{array}$$

DGCS is risk-dominated by DGCN whenever $\gamma_{s}>0$ . They are neutral when $\gamma_{s}=0$ . However, DGCN is always risk-dominated by *D*. Thus, there is a cyclic pattern from DGCS (social guilt), to DGCN (non-social guilt), to *D* (non-emotional defectors) and back to DGCS, whenever the condition in equation (3.3) holds. That occurs when γ and $\gamma_{s}$ are sufficiently small. Fixing c , the latter condition is more easily satisfied for a more beneficial PD (i.e. large b ).

Moreover, for DGCS to be risk-dominant against all the defective strategies (i.e. all but *C* and DGCN), the guilt cost γ needs to be sufficiently large; that is, at least the cost of cooperation, c . Given that, the smaller the social cost, the easier it is for these conditions to be satisfied. The upper bound of this cost is $\frac{\left(\Omega -1)(b-c\right)}{\Omega +1}$ .

### 3.2. Well-mixed populations: evolution of social guilt and the eradication of non-social guilt

To illustrate the above-obtained analytical observations, figure 2 shows the stationary distribution and transition directions in a well-mixed population of the six strategies (see §2). We can see that the directions of transition, showing the risk-dominance of the strategy at the end of the transition or arrow, corroborate the analytical conditions. We further perform replicator dynamics and a brief analysis of equilibrium points in §§1.3 and 1.4 of the electronic supplementary material (see also electronic supplementary material, figures S4 and S5).

**Figure 3.**
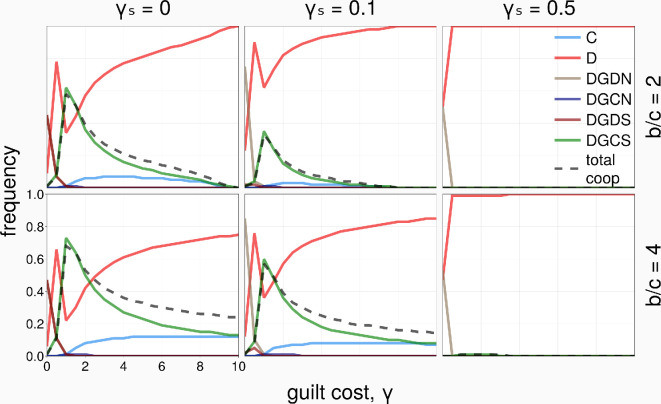
Strategies’ frequency and total cooperation level as a function of the guilt cost, γ (well-mixed, N=100 , Ω=10 ).

Figure 3 shows the long-term frequencies of the strategies and the total level of cooperation in the population, for varying the guilt cost γ , for different benefits b=2 (first row) and b=4 (second row) and for different social costs $\gamma_{s}$ . We observe that, when the social cost $\gamma_{s}$ is sufficiently small, there is an intermediate value of the guilt cost γ (around γ=c ), which leads to an optimal frequency of DGCS and total cooperation in the population. When γ is too small, DGCS is dominated by DGDN (and DGDS; see also figure 2, first column). When γ is larger, *D* frequency increases and dominates the population, despite being still dominated by DGCS (see figure 2, second column). There is now a transition from DGCS to *C*, which is strongly dominated by *D*. Comparing the first and second rows of figure 3, a higher level of cooperation is achieved for a larger benefit of cooperation b .

In short, we can observe that social guilt (DGCS) can evolve in the well-mixed population setting when the social cost is sufficiently small, reaching its peak around γ≈c . Non-social guilt does not evolve at all in this setting, even when it dominates DGCS (whenever $\gamma_{s}>0$ ; see figure 2, second and third rows), as DGCN is always strongly dominated by D.

### 3.3. Structured populations enhance social guilt and enable the emergence of non-social guilt

**Figure 4.**
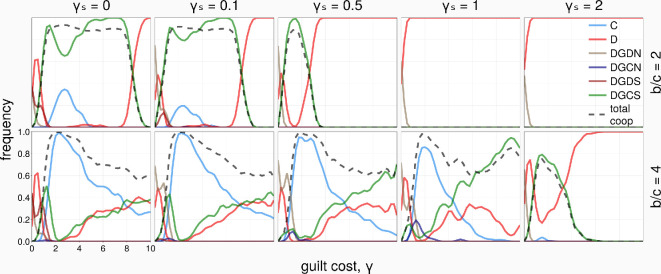
Strategies’ frequency and the total cooperation level as a function of the guilt cost, γ (SL, N=900 , Ω=10 ).

We study the effect of spatial or structured populations on the evolutionary dynamics and outcomes of guilt-prone strategies (both social and non-social), as well as cooperation. Firstly, we consider results in the SL network, a regular (homogeneous) structure (figure 4). We observe that, for a small benefit of cooperation b=2 (top row), for sufficiently small social costs $\gamma_{s}$ (0 and 0.1), DGCS dominates the population over a wide range of γ , between approximately 1<γ<8 . Interestingly, there is also a chance for C to emerge. Moreover, when b is larger (bottom row), C even dominates the population for a wide range of γ and $\gamma_{s}$ . DGCS dominates when γ is sufficiently high. Interestingly, in such networked populations, even non-social guilt strategies can survive with some frequency when the social cost is non-negligible, see $\gamma_{s}=0.1,0.5$ and 1 at intermediate ranges of γ . Overall, we observe significantly higher levels of cooperation and guilt-prone strategies for a wider range of both guilt and social costs, compared to well-mixed populations.

**Figure 5.**
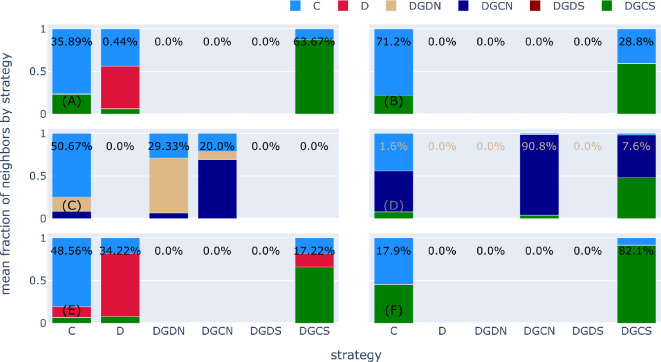
Structured populations foster clustering in mixed-strategy outcomes. The stacked bars represent the mean fraction of strategists in the neighbourhood for each focal strategist. The percentage shown on the bar represents the total fraction of those players in the population. Left column reports results for SL ( N=900 ) and right one for SF networks ( N=1000 ). Typical runs selected to show mixed-strategy outcomes if available (more replicates and different parameter values in the electronic supplementary material). Parameters: Ω=10 ; b=2 , γ=4 , $\gamma_{s}=0$ (A,B); b=4 , γ=1 , $\gamma_{s}=1$ (C,D); b=4 , γ=7 , $\gamma_{s}=0$ (E,F).

Importantly, we see a shift in the cyclic dynamics previously encountered in well-mixed populations. This property can be clarified by observing the clustering behaviours typical of structured populations, even in the case of homogeneous graphs (see figure 5, left column). Typically, we see that unemotional cooperators (*C*) are better protected against unemotional defectors (*D*) when spatiality allows for network reciprocity, especially when evolutionary dynamics lead to mixed-strategy outcomes (no one strategy fully dominates the others). Through such clusters, emotionally adaptive strategists (DGCN and DGCS) can often survive in the face of *D* players. Moreover, this can allow for the co-existence of guilt-prone individuals in communities of other like-minded strategists and *C* players, especially if the cost of being social ( $\gamma_{s}$ ) is low enough (e.g. $\gamma_{s}=0$ and $\gamma_{s}=1$ , as highlighted in figure 5).

We now consider a more complex network structure, the SF network, heterogeneous and highly diverse in the number and distribution of connections. Previous works studying the evolution of cooperation on different networks showed that SF properties can markedly promote cooperation in one-shot social dilemmas, as heterogeneity in the network structure allows cooperators to form clusters around highly connected nodes (hubs) [31,33,52]. Our aim is to study whether this property would also allow pro-social behaviours to evolve; that is, strategies that would not have had a chance to do so previously. To this end, we investigate whether non-social guilt strategies can emerge, leading to even higher levels of (less costly) cooperation overall.

**Figure 6.**
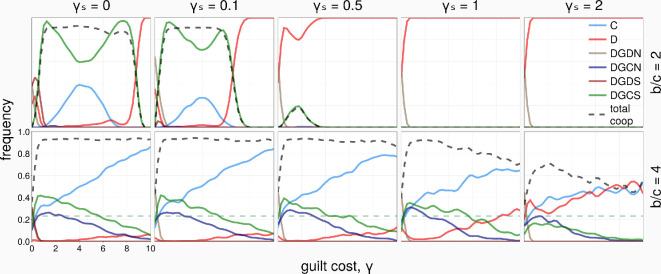
Strategies’ frequency and the total cooperation level as a function of the guilt cost, γ (SF, N=1000 , Ω=10 ). When shown, the dashed green line marks the baseline level of cooperation achieved solely through network reciprocity.

We observe similar outcomes to SL when b=2 , with a slight decrease of cooperation when $\gamma_{s}=0.5$ (figure 6). When b=4 , we find higher levels of cooperation in SF than in SL, across a wide range of guilt and social costs. This improvement can be attributed to the success of non-social guilt, which becomes rather abundant across the entire parameter space. This is a remarkable observation, whereby the easily exploitable non-social individuals (which are nevertheless also desirably cost-efficient) can evolve and co-exist with other strategies in an evolving population/MAS of self-interested agents.

To further explain this finding and confirm our intuitions, we show the clustering behaviours typical of SF populations in figure 5, right column. Given a low social cost $\gamma_{s}$ , social guilt can thrive even in cases when the cost of guilt γ is very large (see figure 5B,F). Communities of emotionally adaptive individuals co-evolve and co-exist, surviving in the face of the predictions of evolutionary dynamics in homogeneous populations. That is, emotionally sacrificial strategies are empowered through heterogeneous environments, even in an incipient form that does not require costly monitoring of the surrounding contexts.

## 4. Discussion

Based on psychological and evolutionary accounts of guilt and social emotions, the present paper studies an evolutionary game theoretical model with social and non-social guilt-prone strategies in co-presence, in the context of differently structured populations (or distributed MAS). The paper considered several important population structures, from homogeneous ones, in the forms of well-mixed and SLs, to heterogeneous, SF networks, showing that the evolutionary outcomes of social and non-social guilt strategies are highly dependent on the underlying population structure. We showed, in the context of the IPD, that only social guilt can evolve in the well-mixed population context, which is in line with previous findings in the literature [10] (see electronic supplementary material for additional analyses where social and non-social guilt strategies are considered separately). Spatial structures, even homogeneous ones (e.g. SLs), allow guilt-prone strategies and cooperation to prevail for a much wider range of the guilt and social costs (compared to the well-mixed setting). Interestingly, heterogeneous networks (i.e. SF), and to a lesser extent SLs, allow non-social guilt to evolve through the formation of clusters with other emotional agents to defend against exploitation. This finding is remarkable, as it showed that costly guilt-prone strategies can prevail in spatial environments, even in an incipient form that does not require expensive monitoring of the context behind others’ actions. This is especially true when the underlying networks mirror realistic, heterogeneous structures [32].

The problems of explaining the evolution and emergence of collective behaviours, such as cooperation, coordination and AI safety in dynamical populations or systems of self-interested agents, have been actively studied across disciplines, from evolutionary biology, physics, economics, to AI and MAS [39,53–66]. Several mechanisms have been proposed to explain the dilemmas of cooperation, including kin selection, direct and indirect reciprocity, incentives or networked structures (see surveys in [27,37,39,67]). In contrast, there is a significant lack of studies looking at the role of cognitive and emotional mechanisms in behavioural evolution [68–72]. Acknowledging the pivotal role of emotions in human decision-making [6,73], it is essential to incorporate these complex mechanisms for a more holistic portrayal of the evolution of cooperation. Our work strives to bridge this gap, providing key insights into the design and engineering of self-organized and distributed MAS, especially in the context of a hybrid human–AI setting, such as cooperative AI [69–71,74–77].

Most relevant to our work is the EGT model proposed in [10], showing that cooperation does not emerge when agents only alleviate their own guilt (i.e. non-social guilt), without considering their co-players’ own attitudes about the alleviation of guilt as well. That is the case where guilt-prone agents are easily dominated by agents who do not express guilt or who have no motivation to alleviate their own guilt. Hence, only when the tendency to alleviate guilt is mutual (i.e. social guilt) can cooperation thrive. This previous work did not consider that choosing to be social might require a cost (compared to being non-social), and thus the latter might have an evolutionary advantage against the former. Indeed, our (risk-dominance) analysis shows that in a direct competition, a non-social guilt strategy is risk-dominant or advantageous against a social one. Because this prior work did not consider both guilt-prone strategies in co-presence within a population, it was not possible to address how this social cost might affect the evolutionary outcomes. The present work considers an extended model where all these strategies are in co-presence together with other non-emotional strategies in a population, so as to address these issues. Moreover, the prior work [10] only focused on the well-mixed population setting, therefore failing to assess how the structure of the underlying network of contacts among the agents in the population affects the evolutionary outcome and the design of cooperative societies. For example, our results show that a spatial structure, even if homogeneous like SLs, allows guilt-prone strategies and cooperation to prevail for a much wider range of the guilt and social costs (compared to the well-mixed setting). Heterogeneous (SF) networks, and to some extent SLs, allow non-social guilt to evolve through clustering of guilt-prone individuals to avoid their exploiters.

Guilt has been considered implicitly in prior EGT models studying apology and forgiveness in social dilemma games [78–81]. These works do not look at guilt as part of agents’ strategies, but rather it plays an implicit role in leading agents to make an apology after wrongdoings. In our present work, the modelling of guilt as a behavioural feature of a strategy enables exploration of new aspects related to feeling guilty, namely its social aspects and how it interacts with external factors, like the network’s structure.

Our modelling work is inspired by a large number of works from psychological, sociological and philosophical literature. Ramsey & Deem [82] argue that the evolutionary emergence of the emotion of guilt needs support for the evolution of empathy. From a multi-agent perspective, including mixed social–technological communities encompassing potentially autonomous artificial agents, and invoking the so-called ‘value alignment’ problem (for a recent review, see [83]). In line with [10], the outcomes from our analyses help confirm that conflicts can be avoided when morally salient emotions, like guilt, help guide participants towards acceptable behaviours. In this context, systems involving possible future artificial moral agents may be designed to include guilt, to align agent-level behaviour with human expectations, thereby resulting in overall social benefits through improved cooperation.

Finally, there exists a large body of computational modelling works of guilt in AI and MAS literature [73,79,84–89]. Unlike our intended outcome, these studies are geared towards the formalization of guilt within MAS, including virtual and cognitive agent systems. The purposes range from regulating social norms [87] to improving agent decision-making and reasoning processes [6,73]. Beyond that, our results provide novel insights into the design and engineering of such MAS systems; for instance, if agents are equipped with the capacity of guilt feeling, even if it might lead to costly disadvantage, that can drive the system to an overall more cooperative outcome where they are willing to take reparative actions after wrongdoings. Additionally, our analysis provides insights on how such guilt-capable agents should be distributed to optimize cooperative outcomes, depending on the specific MAS network structure [6,73,85].

To be evolutionarily viable, an advantageous guilt-prone agent-genotype must act in view of the capacity for its game partners to also express guilt, for a diversity of network structures. The lesson from these experiments is that self-punishment by suffering guilt, without considering whether partners are also similarly guilt-affected, does not result in guilt becoming a dominant advantageous feature of individuals in the population. On the contrary, when defecting partners do not express guilt when agents themselves do, then an agent should either not experience guilt or its guilt should be automatically alleviated, at no cost. Otherwise, guilt-prone agents would be exploited by the non-guilt-prone free-riders with respect to guilt.

Within the IPD, agents assess each other’s actions, deciding whether to defect or cooperate. In real-world scenarios, humans similarly take into account the thought processes that lead others to make these decisions. People, first, tend to trust others who cooperate without ever thinking about defecting over those who do consider defection an option and only later choose against trusting them. According to Kant, ‘In law a man is guilty when he violates the rights of others. In ethics he is guilty if he only thinks of doing so’ [90].

Being attuned to the thought processes or behavioural indications of individuals contemplating cheating or deception entails an added ability to recognize intentions. In accordance with Kant’s insights, Pereira *et al*. [10] affirm that intention recognition plays a crucial role in regulating social interactions, even in cases where a given intention is not explicitly acted upon. However, common sense stresses that feeling guilt for harm done to others only makes sense if one perceives those others did not intend harm and will feel guilty for harm done as well. Where recognizing the intention of another is not considered, then feeling guilty about defections without regard to what others conceivably feel about their defections is self-defeating.

In essence, the present research has provided a robust, game-theoretical-based account of how the interplay between social costs and underlying network structures in a population or distributed MAS enables the co-evolution and co-existence of different types of social and non-social emotions. As a desired result, this strengthens cooperation, though their beholders will incur a significant emotional cost to themselves to achieve this.

## Data Availability

The data, code and supplementary material that support the findings of this study are available at Dryad [91]. Electronic supplementary material is available online [92].

## Footnotes

^1^
There can be other strategies such as emotional (i.e. G=0 ) cooperators who always cooperate and thus never feel guilty. But as we are not modelling noise in this work, this strategy is equivalent to *C* in all interactions and can consequently be removed from our analysis.
